# LincRNA-p21 Inhibits Cell Viability and Promotes Cell Apoptosis in Parkinson's Disease through Activating α-Synuclein Expression

**DOI:** 10.1155/2018/8181374

**Published:** 2018-12-25

**Authors:** Xiaonan Xu, Chengle Zhuang, Zimu Wu, Hongyan Qiu, Haixia Feng, Jun Wu

**Affiliations:** ^1^Department of Neurology, Peking University Shenzhen Hospital, Shenzhen 518000, Guangdong Province, China; ^2^Guangdong and Shenzhen Key Laboratory of Male Reproductive Medicine and Genetics, Institute of Urology, Peking University Shenzhen Hospital, Shenzhen-Peking University-The Hong Kong University of Science and Technology Medical Center, Shenzhen 518000, Guangdong Province, China

## Abstract

Long intergenic noncoding RNA-p21 (lincRNA-p21) has been reported to be increased in Parkinson's disease (PD). However, the function and underlying mechanisms of lincRNA-p21 remain not clear. In order to explore the role of lincRNA-p21 in PD, we used 1-methyl-4-phenyl-1,2,3,6-tetrahydropyridine (MPTP) to induce* in vivo* PD model (C57BL/6 mice) and utilized N-methyl-4-phenylpyridinium (MPP^+^) to create* in vitro* PD model (SH-SY5Y cells). Results showed that the expression level of lincRNA-p21 was increased significantly in PD models. High abundance of lincRNA-p21 inhibited viability and promoted apoptosis markedly in SH-SY5Y cells treated with MPP^+^. Mechanistically, further experiments demonstrated that upregulation of lincRNA-p21 could sponge miR-1277-5p and indirectly increase the expression of *α*-synuclein to suppress viability and activate apoptosis in SH-SY5Y cells. In short, our study illustrated that lincRNA-p21/miR-1277-5p axis regulated viability and apoptosis in SH-SY5Y cells treated with MPP^+^ via targeting *α*-synuclein. LincRNA-p21 might be a novel target for PD.

## 1. Introduction

Parkinson's disease (PD) is a neurodegenerative disease owing to a reduction in dopaminergic neurons in the substantia nigra [1, 2]. The progressive loss of dopamine-producing is the critical pathological hallmark of PD [3]. Myotonia, bradykinesia, depression, anxiety, static tremors, and cognitive dysfunction are typical symptoms of PD [4]. Various factors such as heredity, environment, and life style are closely related to the occurrence of PD, and the prevalence of PD all over the world is 1-2% of populations among populations over 65 years of age [5, 6]. Despite huge attempts to investigate the molecular mechanisms of occurrence of PD, novel targeted drugs are missing and now there is still a lack of known cure for PD [7].

Long noncoding RNAs (lncRNAs) with length of more than 200 nucleotides are involved in various biological processes, such as tumor development, transcriptional regulation, and cell apoptosis [8]. Long intergenic noncoding RNA-p21 (lincRNA-p21), 3100 nt, is located on chromosome 6, involved in cell proliferation, metabolism, and reprogramming, and regarded as a potential diagnostic marker in various diseases [9]. LincRNA-p21, a p53-dependent transcriptional target gene, functions as a transcriptional repressor and triggers cell apoptosis [10]. A study has shown that lincRNA-p21 promotes cell apoptosis in hepatocellular carcinoma through inducing ER stress [11]. LincRNA-p21 plays a suppressive role in prostate cancer through modulating p53 [12]. LincRNA-p21 can activate hepatic stellate cells through lincRNA-p21-miR-181b-PTEN pathway in liver fibrosis [13]. However, the function of this lncRNA in PD is still unknown.

The expression levels of different lncRNAs were illustrated in 30 brain specimens derived from patients diagnosed with PD, and lincRNA-p21, SNHG1, MALAT1, and TncRNA expression levels were increased significantly [14]. *α*-Synuclein is natively unfolded protein associated with Parkinson's disease (PD) [15]. After overexpressing *α*-synuclein in the substantia nigra pars compacta of presymptomatic mice, 756 lncRNAs were expressed differently via microarray analysis [16]. Ye et al. reported that lincRNA-p21 sponged miR-181 promoted microglial activation through enhancing the expression of PKC-*δ* in PD models [17]. However, more indepth researches are needed to investigate the role of lincRNA-p21 and its pathogenic mechanism of PD.

In this study, we investigate the effect of lincRNA-p21 on cell viability and apoptosis in PD model cells (SH-SY5Y) treated with N-methyl-4-phenylpyridinium (MPP+). Besides, further mechanism of lincRNA-p21 on cell viability and apoptosis was manifested. Our work may provide another theoretical basis that lincRNA-p21 might be a novel target for PD.

## 2. Materials and Methods

### 2.1. Animals

Male C57BL/6 mice (5-10 weeks old) were purchased from Beijing Vital River Laboratory Animal Technology Co., Ltd., Beijing, China. 12 mice were randomly divided into two groups equally: the negative control group and the 1-Methyl-4-phenyl-1,2,3,6-tetrahydropyridine (MPTP) group. Sterile saline solution was injected intraperitoneally with a dose of 20 mg/kg body weight (four times/per day, two hour intervals) in negative control group. MPTP (Sigma, St. Louis, MO, USA) was injected intraperitoneally with an equal volume of sterile saline solution in MPTP group. The midbrains were isolated and harvested for the subsequent analysis after sacrifice with the last MPTP injection in the 21st day. The experiments procedures were approved by the Institutional Ethics Review Board of Peking University Shenzhen Hospital and were carried out according to the Guide for Care and Use of Laboratory Animals.

### 2.2. Cell Culture

SH-SY5Y cell line (human neuroblastoma cells) was purchased from American Type Culture Collection (ATCC) (Manassas, Va., USA). The SH-SY5Y cell line was cultured according to the suggestions of ATCC. SH-SY5Y was pretreated with 100 *μ*M 1-Methyl-4-phenylpyridine (MPP^+^) (Sigma) for 24 hours to get the* in vitro* PD model.

### 2.3. Plasmid Construction and Cell Transfection

The sequence of lincRNA-p21 was synthesized by GenePharma Co. Ltd. (Shanghai, China) and then cloned into pcDNA3.1 vector between the XhoI and BamHI sites to create the pcDNA3.1-lincRNA-p21 plasmid, and then the CMV promoter derived the expression of lincRNA-p21. The siRNAs targeting lincRNA-p21 or *α*-synuclein were synthesized by GenePharma Co. Ltd. (Shanghai, China). The miR-1277-5p mimics and inhibitors were purchased from GeneCopoeia Co. Ltd. (Guangzhou, China). The sequence of 3′ UTR of *α*-synuclein or mutant 3′ UTR of *α*-synuclein was inserted into 3′ UTR of renilla luciferase gene digested with Xhol and Notl in the psiCHECKTM-2 luciferase reporter plasmid purchased from Promega, Madison, WI, USA. Plasmids, siRNAs, or miR-1277-5p mimics/inhibitors were transfected into SH-SY5Y cells using Lipofectamine 3000 (Invitrogen, Thermo Fisher Scientific, Inc.) according to the manufacturer's protocols. All experiments were repeated at least three times.

### 2.4. Quantitative Real-Time Polymerase Chain Reaction (qRT-PCR)

The TRIzol reagent (Invitrogen, Grand Island, NY, USA) was used to extract total RNA from mice tissues or cells after transfection according to the manufacturer's protocol. The first strand of cDNA for detection of lincRNA-p21 and *α*-synuclein was synthesized using PrimeScript RT Reagent Kit with gDNA Eraser (Takara, Dalian, China). The qRT-PCR for detection of lincRNA-p21 and *α*-synuclein was carried out utilizing the SYBR Premix Ex Taq^TM^ II (Takara, Dalian, China) in the Roche LightCycler 480 Real-Time PCR System. GAPDH was used as the endogenous control to normalize the data of lincRNA-p21 and *α*-synuclein. The cDNA for detection of miR-1277-5p was synthesized using All-in-One miRNA First-Strand cDNA Synthesis Kit (GeneCopoeia Co. Ltd., Guangzhou, China), and qRT-PCR for it was performed using All-in-One miRNA qRT-PCR Detection Kit (GeneCopoeia Co. Ltd., Guangzhou, China). The expression of miR-1277-5p was normalized using U6-snRNA as internal control. The primers were lincRNA-p21-forward: 5′- GAAAGCGAGTGGGACAGG -3′, lincRNA-p21-reverse: 5′-CAGGGCAAGAACTTGTGGAC-3′; *α*-synuclein-forward: 5′-AAGAGGGTGTTCTCTATGTAGGC-3′, *α*-synuclein-reverse: 5'-GCTC CTCCAACATTTGTCACTT-3′; GAPDH-forward: 5′-CGCTCTCTGCTCCTCCTG TTC-3′, GAPDH-reverse: 5′-ATCCGTTGACTCCGACCTTCAC-3′. Primers of miR-1277-5p were purchased from GeneCopoeia Co. Ltd., Guangzhou, China. The 2^-ΔΔCt^ method was used to analyze the relative expression of lincRNA-p21, miR-1277-5p, and *α*-synuclein. All experiments were performed in triplicate.

### 2.5. Cell Nucleus and Cytoplasm Fraction Isolation

NE-PER Nuclear and Cytoplasmic Extraction Reagents (Thermo Scientific, Waltham, MA, USA) were used to prepare cytoplasmic and nuclear extracts from SH-SY5Y cells pretreated with MPP^+^. RNAs separated from each of the fractions were used for qRT-PCR analysis to illustrate the levels of nuclear control transcript (U6), cytoplasmic control transcript (GAPDH), and lincRNA-p21.

### 2.6. Western Blot Analysis

The protein concentration was measured by the bicinchoninic acid (BCA) quantification assay (Pierce Biotechnology, Rockford, IL, USA). Equal amounts (20 *μ*g) of whole protein extract were electrophoresed on SDS-polyacrylamide gels and transferred to polyvinylidene difluoride membranes using a semidry transfer cell (Bio-Rad Laboratories, Hercules, CA, USA). The membranes were incubated overnight at 4°C with specific primary antibodies against *α*-synuclein (1:1000; Abcam, USA) and *β*-actin (1:1000; Abcam, USA) after being blocked with 5% nonfat dry milk to prevent the nonspecific binding of the primary antibody. Horseradish peroxidase (HRP) conjugated secondary antibody (1:10000; Amersham, Arlington Heights, IL) was used to incubate the blot for one hour at room temperature on a rocking platform. Besides, the blot was washed with 1x TBST three times. Finally, super signal chemiluminescence reagents (Thermo Fisher Scientific, Inc., Massachusetts, USA) were used to detect signal intensities.

### 2.7. RNA Immunoprecipitation (RIP) Assay

RIP assays were carried out in SH-SY5Y cells pretreated with MPP^+^, 48 hours after transfection with miR-1277-5p or miR-NC, utilizing the Magna RIP RNA-binding protein immunoprecipitation kit (Millipore, Billerica, MA, USA). Cells (1 × 10^7^) were lysed in complete RNA lysis buffer, then cell lysates were incubated with anti-Argonaute 2 (AGO2) antibody (Millipore, Billerica, Ma, USA), negative control mouse IgG (Millipore, Billerica, Ma, USA) or total RNAs (input controls) in accordance with the manufacturer's instructions. Coprecipitated RNA was measured by qRT-PCR as previously described.

### 2.8. Dual-Luciferase Activity Assay

SH-SY5Y cells were seeded in 24-well plates (1 × 10^5^ per well). SH-SY5Y cells were transfected with psi-*α*-synuclein or mutant *α*-synuclein psi-mut-*α*-synuclein, along with miR-1277-5p mimics or inhibitors utilizing transfection reagent (Invitrogen). After 48-hour transfection, luciferase activity was measured through the dual-luciferase assay system (Promega. Madison, WI, USA) in accordance with the manufacturer's instructions. To calculate the activity of luciferase, the activity of renilla luciferase was normalized to the activity of firefly luciferase in psiCHECKTM-2 vector.

### 2.9. Cell Viability Assay

The cell viability was measured using Cell Counting Kit-8 (CCK-8; Beyotime Institute of Biotechnology, Shanghai, China). 5 × 10^3^ SH-SY5Y cells per well were seeded into 96-well plates. 10 *μ*l of CCK-8 reagent was added to each well and then incubated at 37°C for 2 hours. Absorbance at a wavelength of 450 nm was detected by a microplate reader (Bio-Rad, Hercules, CA, USA) following the manufacturer's protocols. Each test was performed at least three times.

### 2.10. Cell Apoptosis Assay

Cells were seeded in 12-well plates and transfected with plasmid, siRNAs or miRNAs. The activity of caspase-3 represented the levels of apoptosis and was detected employing the caspase-3 enzyme-linked immunosorbent assay (ELISA) kit (Cusabio, Wuhan, China) according to the manufacturer's protocol. And this assay was widely used to detect cell apoptosis [18]. All experiments were carried out at least three times.

### 2.11. Statistical Analysis

All experimental data from three independent experiments were presented as mean ± standard deviation (SD) and processed by SPSS 19.0 software (SPSS Inc., Chicago, IL, USA). Student's* t-*test was used to analyze the difference of two groups. For more than two groups, the difference was analyzed by one-way ANOVA followed by a post hoc Tukey's test. Difference was considered statistically significant when a two-tailed value of* P* < 0.05 was exhibited.

## 3. Results

### 3.1. Overexpression of LincRNA-p21 Inhibited Cell Viability and Induced Apoptosis

**Figure 1:**
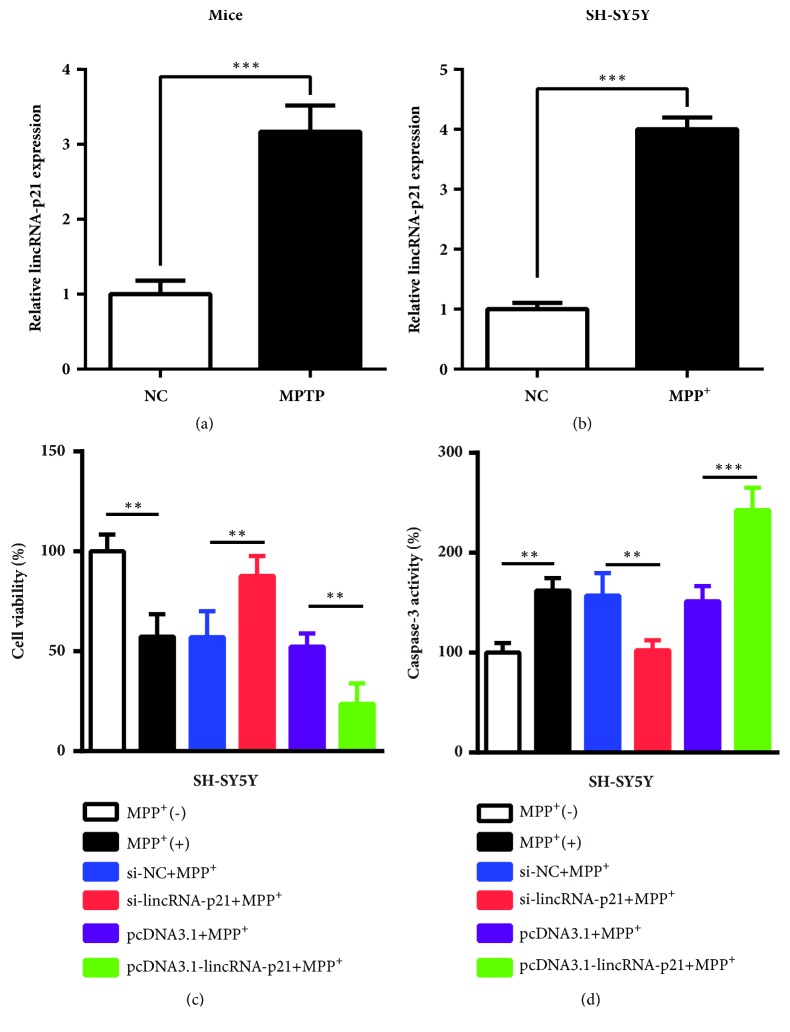
**The expression levels of lincRNA-p21 was detected in PD model mice and cells, and effects of lincRNA-p21 on cell viability and apoptosis of PD cells were shown**. (a, b) The expression levels of lincRNA-p21 were increased significantly in PD mice and MPP^+^-induced SH-SY5Y cells. (c) Knockdown or overexpression of lincRNA-p21 increased or suppressed cell viability obviously in SH-SY5Y cells treated with MPP^+^. (d) Knockdown or overexpression of lincRNA-p21 inhibited or increased the activity of caspase-3 (cell apoptosis) markedly in SH-SY5Y cells treated with MPP^+^. All data are shown as the mean ± SD (*∗∗*P < 0.01, *∗∗∗*P < 0.001); bar, SD.

Compared with the corresponding negative control, the expression levels of lincRNA-p21 were increased significantly in PD mice and* in vitro* PD model SH-SY5Y (Figures 1(a) and 1(b)). Then, knockdown and overexpression of lincRNA-P21 were performed through siRNAs and overexpression plasmid pcDNA3.1. As shown in Figure 1(c), cell viability was decreased significantly in only MPP^+^ group (P < 0.01) compared with the untreated with MPP^+^ group. After knockdown of lincRNA-p21 in SH-SY5Y cells treated with MPP^+^, cell viability was increased remarkably compared with the si-NC+ MPP^+^ group (P < 0.01). After overexpression of lincRNA-p21 in SH-SY5Y cells treated with MPP^+^, cell viability was reduced obviously compared with the pcDNA3.1+ MPP^+^ (P < 0.01). Next, the effects of lincRNA-p21 on cell apoptosis were also measured. As presented in Figure 1(d), compared with the untreated SH-SY5Y cells, the caspase-3 activity was improved significantly in SH-SY5Y cells treated with MPP^+^ (P < 0.01). Compared with si-NC+ MPP^+^ group, the activity of caspase-3 was inhibited dramatically in si-lincRNA-p21 group+ MPP^+^ group (P < 0.01). However, after overexpression of lincRNA-p21, cell apoptosis was increased significantly in cells treated with MPP^+^ (P < 0.001). Thus, high abundance of lincRNA-p21 suppressed cell viability and promoted cell apoptosis in PD models.

### 3.2. LincRNA-p21 Sponged miR-1277-5p

**Figure 2:**
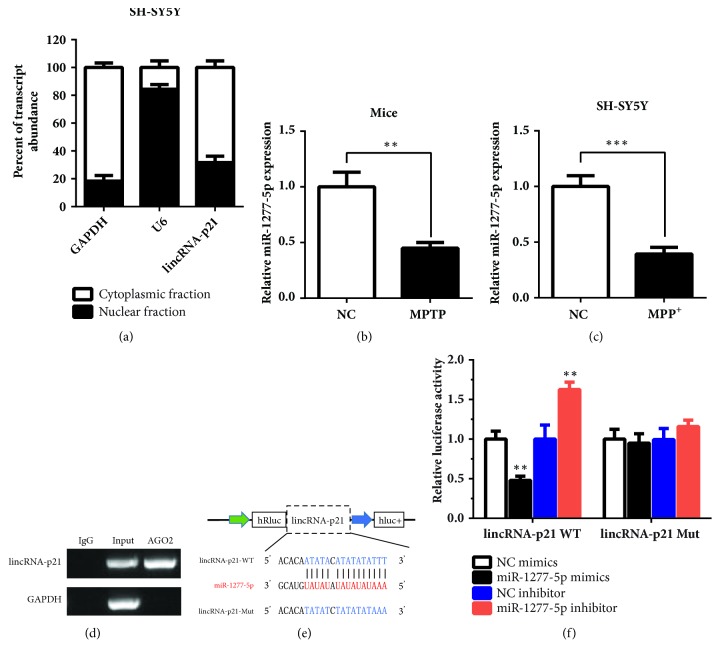
**The miR-1277-5p was sponged by lincRNA-p21**. (a) LincRNA-p21 was mainly located in cytoplasm. (b, c) The expression of miR-1277-5p was downregulated significantly in PD mice and PD model cells, SH-SY5Y. (d) RIP was performed and coprecipitated RNA was utilized for PCR assay. IgG, negative control group; input, total RNA group; AGO2, AGO2 antibody group. (e) The sequence of wide-type lincRNA-p21 or mutated lincRNA-p21 was inserted into the 3′ UTR of renilla luciferase (hRluc). (f) The luciferase activity was decreased significantly after transfection of miR-1277-5p mimics in lincRNA-p21 WT group. The luciferase activity was increased obviously after transfection of miR-1277-5p inhibitor in lincRNA-p21 WT group. However, there was no difference in lincRNA-p21 Mut group. All data are presented as the mean ± SD (*∗∗*P < 0.01, *∗∗∗*P < 0.001); bar, SD.

In order to investigate the mechanisms of lincRNA-p21 in PD, we firstly tested the subcellular localization of lincRNA-p21. As shown in Figure 2(a), lincRNA-p21 was mainly located in cytoplasm. Thus, lincRNA-p21 may inhibit cell viability and apoptosis at posttranscriptional level. Next, we speculated that lincRNA-p21 may sponge miRNAs. According to the miRDB, TargetScan, and miRWalk database, miR-1277-5p can bind to lincRNA-p21. Then, qRT-PCR data showed that the expression of miR-1277-5p was decreased significantly in PD mice (Figure 2(b), P < 0.01) and SH-SY5Y cells treated with MPP^+^ (Figure 2(c), P < 0.001). To verify whether lincRNA-p21 sponged miR-1277-5p, we performed RIP and dual-luciferase assays. It has been known that miRNAs degrade mRNA and inhibit translation in an Argonaute 2 (AGO2)-dependent manner by binding to their targets [19]. We conducted anti-AGO2 RIP in SH-SY5Y cells transiently overexpressing miR-1277-5p to pull down lincRNA-p21 using control IgG or AGO2 antibodies, followed by PCR analysis for lincRNA-p21 levels. As presented in Figure 2(d), compared with the anti-IgG control, lincRNA-p21 pulled down with anti-AGO2 antibodies was enriched obviously in SH-SY5Y cells transfected with miR-1277-5p mimics. However, GAPDH was not pulled down with anti-AGO2 antibodies. This result suggested that miR-1277-5p could directly target lincRNA-p21 in AGO2 manner. Besides, the wide-type lincRNA-p21 sequence (WT) or the sequence with mutated binding sites of miR-1277-5p (Mut) was inserted into the 3′ UTR of the renilla luciferase in ps-CHECK2 vector (Figure 2(e)). As shown in Figure 2(f), after overexpressing of miR-1277-5p, the luciferase activities of WT reporter were decreased significantly compared with the Mut reporter. On the contrary, the luciferase activities of WT reporter were increased remarkably compared with the Mut control after inhibition of expression of miR-1277-5p. These results demonstrated that lincRNA-p21 suppressed the activity of miR-1277-5p via direct binding between them and lincRNA-p21 might be regarded as a competing endogenous RNA (ceRNA) for miR-1277-5p.

### 3.3. *α*-Synuclein Was a Target Gene of miR-1277-5p

**Figure 3:**
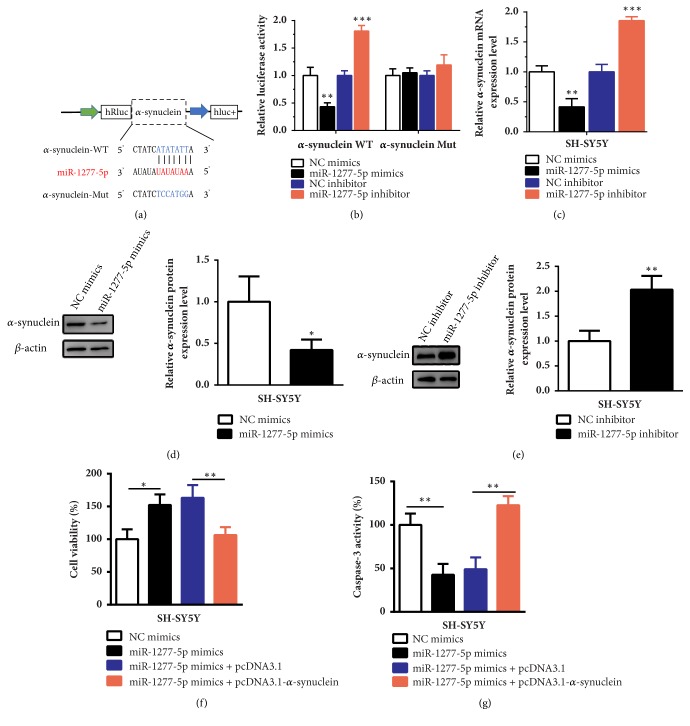
α**-synuclein was the target gene of miR-1277-5p**. (a) The sequence of wide-type *α*-synuclein or mutated *α*-synuclein was inserted into the 3′ UTR of renilla luciferase (hRluc). (B) The luciferase activity was suppressed significantly after transfection of miR-1277-5p mimics in *α*-synuclein WT group. The luciferase activity was stimulated markedly after transfection of miR-1277-5p inhibitor in *α*-synuclein WT group. However, there was no difference in *α*-synuclein Mut group. (c, d, e) The *α*-synuclein mRNA and protein expression levels were measured after transfected with miR-1277-5p mimics or inhibitor. (f, g) The rescue effects of *α*-synuclein on cell viability and apoptosis after overexpression of miR-1277-5p in SH-SY5Y cells treated with MPP^+^ were presented. All data are shown as the mean ± SD (*∗*P < 0.05, *∗∗*P < 0.01, *∗∗∗*P < 0.001); bar, SD.

According to the online database TargetScan (http://www.targetscan.org), *α*-synuclein is a target gene of miR-1277-5p. In order to verify whether miR-1277-5p bound to *α*-synuclein, we performed the luciferase assay. The wide-type *α*-synuclein sequence (WT) or the sequence with mutated binding sites of miR-1277-5p (Mut) was inserted into the 3′ UTR of the renilla luciferase in ps-CHECK2 vector (Figure 3(a)). As shown in Figure 3(b), after cotransfecting with *α*-synuclein WT luciferase reporter and miR-1277-5p mimics, the activity of luciferase was decreased significantly compared with the NC mimics group (P < 0.01). However, the luciferase activity was increased remarkably after cotransfecting with *α*-synuclein WT luciferase reporter and miR-1277-5p inhibitor (P < 0.001). Besides, there was no difference in *α*-synuclein Mut group. Next, we tested the effects of miR-1277-5p on the mRNA and protein expression levels of *α*-synuclein. After transfection of miR-1277-5p mimics, the *α*-synuclein mRNA expression level was decreased significantly compared with the NC mimics group (Figure 3(c), P < 0.01). However, *α*-synuclein mRNA expression level was increased remarkably after transfection of miR-1277-5p inhibitor in SH-SY5Y cells (Figure 3(c), P < 0.001). Similarly, *α*-synuclein protein expression level was suppressed obviously in miR-1277-5p mimics group compared with the NC mimics group (Figure 3(d), P < 0.05). Conversely, the *α*-synuclein was upregulated significantly after inhibition of miR-1277-5p in SH-SY5Y cells treated with MPP^+^ (Figure 3(e), P < 0.01). In a word, above results indicated that *α*-synuclein was the target gene of miR-1277-5p in PD model.

### 3.4. The High Expression of *α*-Synuclein Can Abrogate the Protective Effects of miR-1277-5p in PD Model SH-SY5Y Cells

As shown in Figure 3(f), after transfection of miR-1277-5p mimics in SH-SY5Y cells, cell viability was increased significantly compared with NC mimics group (P < 0.05). However, cell viability was decreased remarkably after cotransfection of miR-1277-5p mimics and *α*-synuclein overexpression plasmid (pcDNA3.1-*α*-synuclein) (P < 0.01). Overexpression of miR-1277-5p could inhibited caspase-3 activity dramatically (Figure 3(g), P < 0.01). But after cotransfection of miR-1277-5p and pcDNA3.1-*α*-synuclein in SH-SY5Y cells, the activity of caspase-3 was promoted obviously compared with the corresponding negative control (Figure 3(g), P < 0.01). These results demonstrated that miR-1277-5p promoted cell viability and inhibited cell apoptosis in PD model SH-SY5Y cells. Nonetheless, overexpression of *α*-synuclein could abolish the effects of miR-1277-5p in SH-SY5Y cells.

### 3.5. LincRNA-p21 Regulated *α*-Synuclein via miR-1277-5p

**Figure 4:**
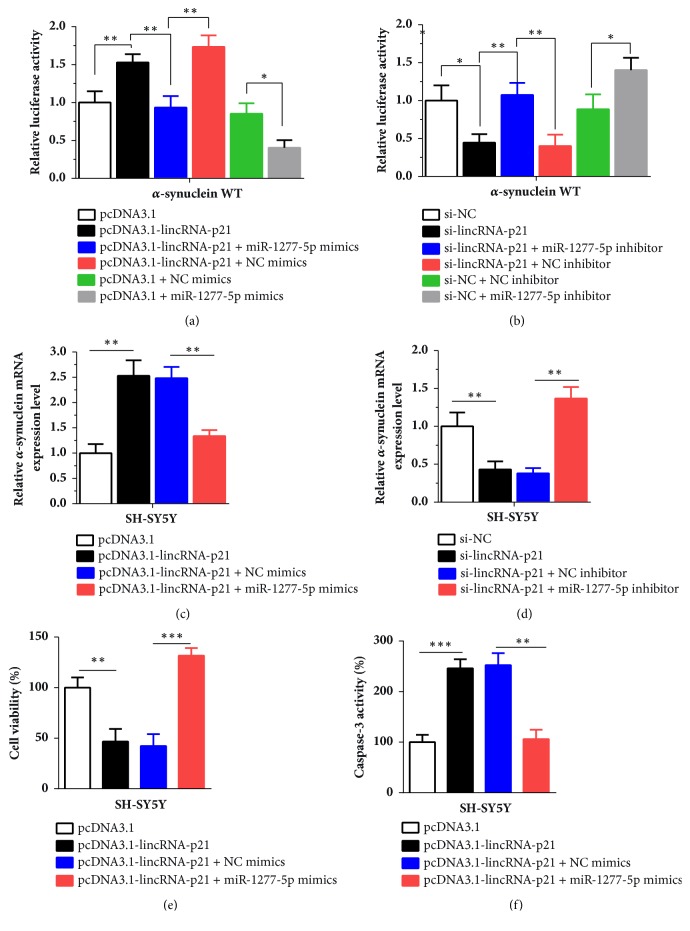
**LincRNA-p21 and miR-1277-5p regulated **α**-synuclein expression and effects of coexpressing lincRNA-p21 and miR-1277-5p mimics on cell viability and apoptosis were detected in SH-SY5Y cells treated with MPP**^**+**^. (a, b) The luciferase activity was detected after cotransfection of lincRNA-p21 and miR-1277-5p mimics/inhibitor in *α*-synuclein group. (c, d) The expression of *α*-synuclein was measured after cotransfection of lincRNA-p21 and miR-1277-5p mimics/inhibitor. (e) Cell viability was measured after cotransfection of lincRNA-p21 and miR-1277-5p mimics in SH-SY5Y cells. (f) Cell apoptosis was observed after cotransfection of lincRNA-p21 and miR-1277-5p mimics in SH-SY5Y cells. All data are shown as the mean ± SD (*∗*P < 0.05, *∗∗*P < 0.01, *∗∗∗*P < 0.001); bar, SD.

In order to further investigate the molecular mechanisms of lincRNA-p21, we transfected the luciferase reporter of WT *α*-synuclein, and pcDNA3.1-lincRNA-p21 and miR-1277-5p mimics into SH-SY5Y cells. As shown in Figure 4(a), results showed that the luciferase activity was increased significantly in pcDNA3.1-lincRNA-p21 group compared with the pcDNA3.1 group (P < 0.01). When cells were cotransfected with pcDNA3.1-lincRNA-p21 and miR-1277-5p, the luciferase activity was decreased remarkably compared with the pcDNA3.1-lincRNA-p21 and NC mimics group (P < 0.01). When cells were cotransfected with pcDNA3.1 and miR-1277-5p mimics, the luciferase was inhibited significantly compared with pcDNA3.1+NC mimics group (P < 0.05). Conversely, as presented in Figure 4(b), the luciferase activity was suppressed obviously after inhibition of lincRNA-p21 (P < 0.05). After inhibition of lincRNA-p21 and miR-1277-5p, the luciferase activity was increased significantly compared with the corresponding negative group (P <0.01). After cotransfection of si-NC and miR-1277-5p inhibitor, the luciferase activity was upregulated evidently (P < 0.05). These results indicated that miR-1277-5p mimics could counteract the luciferase activity. Besides, we verified the effects of lincRNA-p21 and miR-1277-5p on the *α*-synuclein mRNA expression level. Data showed that overexpression of lincRNA-p21 could increase significantly the *α*-synuclein mRNA expression level (Figure 4(c), P < 0.01). However, after overexpression of lincRNA-p21 and miR-1277-5p at the same time, the *α*-synuclein mRNA expression level was decreased evidently compared with the pcDNA3.1-lincRNA-p21+NC mimics group (Figure 4(c), P < 0.01). As presented in Figure 4(d), inhibition of lincRNA-p21 restrained the expression of *α*-synuclein (P < 0.01). When SH-SY5Y cells were transfected with si-lincRNA-p21 and miR-1277-5p inhibitor concurrently, the *α*-synuclein mRNA expression level was increased significantly compared with the si-lincRNA-p21+NC inhibitor group (P < 0.01). Furthermore, overexpression of lincRNA-p21 caused the inhibition of cell viability, but this effect could be counteracted significantly by overexpression of miR-1277-5p (Figure 4(e), P < 0.001). The ELISA assay demonstrated that overexpression of lincRNA-p21 led to increased caspase-3 activity remarkably in SH-SY5Y cells (Figure 4(f), P < 0.001). However, the effect of lincRNA-p21 on the caspase-3 activity was decreased notably by overexpression of miR-1277-5p (Figure 4(f), P < 0.01). These results suggested that lincRNA-p21 could regulate the expression of *α*-synuclein via miR-1277-5p.

## 4. Discussion

Long noncoding RNAs (lncRNAs) participate in the regulation of gene expression, affecting neural plasticity, development, and aging [20, 21]. LncRNAs have been illustrated to function as mediators in miRNAs, mRNA degradation, and protein function [22]. 1-methyl-4-phenyl-1,2,3,6-tetrahydropyridine (MPTP) has been utilized to create PD animal models and 1-methyl-4-phenylpyridine (MPP^+^) was used to establish* in vitro *PD cell model [23, 24]. LincRNA-p21 was one kind of lncRNAs and, in this study, results indicated that the expression of lincRNA-p21 was increased significantly in MPTP-induced PD mice and SH-SY5Y cells treated with MPP^+^, which was consistent with the previous study [14]. This data suggested that lincRNA-p21 may play vital roles in the regulation of neurons cells in PD. Results of cell viability and apoptosis assays showed that high abundance of lincRNA-p21 inhibited viability and induced apoptosis notably in SH-SY5Y cells treated with MPP^+^. Thus, the underlying mechanism of lincRNA-p21 was further investigated in this study.

The miRNA degraded mRNA through binding to the 3′ UTR of mRNA completely and inhibited translation processes via incomplete complementarity between miRNA and downstream mRNA [25]. Various studies demonstrated that miRNAs played vital roles in the pathogenesis of PD [26]. High abundance of lncRNA SNHG1 promoted cytotoxicity and ROS production via miR-15p/ GSK3*β* axis in SH-SY5Y cells treated with MPP^+^ [27]. We suspected that lincRNA-p21 may take effect in PD through miRNA. Our results suggested that lincRNA-p21 was mainly located in cytoplasm, and through bioinformatic analysis, we found that lincRNA-p21 may bind to miR-1277-5p. RIP assay and dual-luciferase assay results verified that lincRNA-p21 bound to miR-1277-5p and regulated the expression of miR-1277-5p. Further experiments indicated that overexpression of miR-1277-5p could abrogate the effects of lincRNA-p21 on cell viability and apoptosis.

PD is one kind of “synucleinopathies” and neurodegenerative diseases [28]. The *α*-synuclein protein of 140 amino acids with an incompletely defined function was involved in synaptic vesicle trafficking [29]. The *α*-synuclein has been verified as a hallmark and critical pathogenic protein in PD [30]. High abundance of *α*-synuclein has developed the PD-like behavioral abnormalities [31]. In this study, we found that *α*-synuclein was the target gene of miR-1277-5p through bioinformatic analysis. Results of dual-luciferase assay, qRT-PCR, and western blot assay proved that *α*-synuclein was really the downstream gene of miR-1277-5p. Effects of miR-1277-5p and *α*-synuclein on cell viability and apoptosis were also measured, and data showed that effects of high expression of miR-1277-5p on cell viability and apoptosis were counteracted by overexpression of *α*-synuclein. Besides, in order to illustrate the relationship among lincRNA-p21, miR-1277-5p, and *α*-synuclein, we also carried out dual-luciferase assay and qRT-PCR to measure the effects on the expression of *α*-synuclein between lincRNA-p21 and miR-1277-5p. Data indicated that high expression of lincRNA-p21 promoted the expression of *α*-synuclein. The expression level of *α*-synuclein was inhibited and abrogated after cotransfection of lincRNA-p21 and miR-1277-5p mimics. Cell viability was inhibited by overexpression of lincRNA-p21. However, cell viability of SH-SY5Y cells treated with MPP^+^ was increased after cotransfection of lincRNA-p21 and miR-1277-5p mimics. Similarly, cell apoptosis was promoted by high expression of lincRNA in SH-SY5Y cells. But after transfection of lincRNA-p21 and miR-1277-5p simultaneously, apoptosis was suppressed significantly. These results demonstrated effects of lincRNA-p21 on cell viability and apoptosis through regulating *α*-synuclein.

## 5. Conclusion

Our study illustrated that lincRNA-p21 inhibited viability and promoted apoptosis of SH-SY5Y cells induced by MPP^+^ via downregulation of miR-1277-5p and upregulation of *α*-synuclein protein. LincRNA-p21 might be regarded as a promising target for PD.

## Data Availability

The data used to support the findings of this study are available from the corresponding author upon request.
